# Neural Representation Enhanced for Speech and Reduced for Background Noise With a Hearing Aid Noise Reduction Scheme During a Selective Attention Task

**DOI:** 10.3389/fnins.2020.00846

**Published:** 2020-09-10

**Authors:** Emina Alickovic, Thomas Lunner, Dorothea Wendt, Lorenz Fiedler, Renskje Hietkamp, Elaine Hoi Ning Ng, Carina Graversen

**Affiliations:** ^1^Eriksholm Research Centre, Oticon A/S, Snekkersten, Denmark; ^2^Department of Electrical Engineering, Linkoping University, Linköping, Sweden; ^3^Department of Health Technology, Technical University of Denmark, Lyngby, Denmark; ^4^Department of Behavioral Sciences and Learning, Linkoping University, Linköping, Sweden; ^5^Oticon A/S, Smørum, Denmark

**Keywords:** hearing impairment, hearing aids, noise reduction scheme, electroencephalography, stimulus reconstruction

## Abstract

**Objectives:**

Selectively attending to a target talker while ignoring multiple interferers (competing talkers and background noise) is more difficult for hearing-impaired (HI) individuals compared to normal-hearing (NH) listeners. Such tasks also become more difficult as background noise levels increase. To overcome these difficulties, hearing aids (HAs) offer noise reduction (NR) schemes. The objective of this study was to investigate the effect of NR processing (inactive, where the NR feature was switched off, *vs.* active, where the NR feature was switched on) on the neural representation of speech envelopes across two different background noise levels [+3 dB signal-to-noise ratio (SNR) and +8 dB SNR] by using a stimulus reconstruction (SR) method.

**Design:**

To explore how NR processing supports the listeners’ selective auditory attention, we recruited 22 HI participants fitted with HAs. To investigate the interplay between NR schemes, background noise, and neural representation of the speech envelopes, we used electroencephalography (EEG). The participants were instructed to listen to a target talker in front while ignoring a competing talker in front in the presence of multi-talker background babble noise.

**Results:**

The results show that the neural representation of the attended speech envelope was enhanced by the active NR scheme for both background noise levels. The neural representation of the attended speech envelope at lower (+3 dB) SNR was shifted, approximately by 5 dB, toward the higher (+8 dB) SNR when the NR scheme was turned on. The neural representation of the ignored speech envelope was modulated by the NR scheme and was mostly enhanced in the conditions with more background noise. The neural representation of the background noise was modulated (i.e., reduced) by the NR scheme and was significantly reduced in the conditions with more background noise. The neural representation of the net sum of the ignored acoustic scene (ignored talker and background babble) was not modulated by the NR scheme but was significantly reduced in the conditions with a reduced level of background noise. Taken together, we showed that the active NR scheme enhanced the neural representation of both the attended and the ignored speakers and reduced the neural representation of background noise, while the net sum of the ignored acoustic scene was not enhanced.

**Conclusion:**

Altogether our results support the hypothesis that the NR schemes in HAs serve to enhance the neural representation of speech and reduce the neural representation of background noise during a selective attention task. We contend that these results provide a neural index that could be useful for assessing the effects of HAs on auditory and cognitive processing in HI populations.

## Summary

Selectively attending to a target talker while ignoring multiple interferers is more difficult for hearing-impaired (HI) individuals compared to their normal-hearing (NH) peers and becomes more difficult with increased background noise levels. To overcome such difficulties, hearing aids (HAs) offer noise reduction (NR) schemes. Here we aimed to investigate the effect of NR processing on the neural representation of speech envelopes across two different background noise levels by using a stimulus reconstruction (SR) method. The electroencephalogram was recorded while 22 HI participants fitted with HAs were performing a listening task. The participants were instructed to listen to a target talker in front of them while ignoring a competing talker also in front in the presence of multi-talker background babble noise. Measures of neural representation for the envelopes of the attended speech, the unattended speech, and the background noise were calculated separately. The results show that the neural representation of the attended speech envelope was enhanced by the active NR scheme for both background noise levels. The neural representation of the attended speech envelope at low signal-to-noise ratio (SNR) was shifted, approximately by 5 dB, toward the higher SNR when the NR scheme was turned on. The neural representation of the ignored speech envelope was modulated by the NR scheme and was mostly enhanced in the conditions with more background noise. The neural representation of the background noise was modulated by the NR scheme and was significantly reduced in the conditions with more background noise. The neural representation of the net sum of the ignored acoustic scene (ignored talker and background babble) was not modulated by NR scheme but was significantly reduced in the conditions with a reduced level of background noise. We conclude that, during a selective attention speech task, the active NR scheme enhanced the neural representation of both the attended and the ignored speakers and reduced the neural representation of background noise, while the net sum of the ignored acoustic scene was not enhanced. We contend that these results provide a neural index that could be useful for assessing the effects of HAs on auditory and cognitive processing in HI populations.

## Introduction

Natural speech communication with multiple concurrent talkers requires a listener to be able to selectively attend to a talker while ignoring interfering talkers or background noise (Cherry, 1953; Fritz et al., 2007). Behavioral studies have shown that this ability to segregate multiple talkers and selectively attend to a particular talker is decreased in hearing-impaired (HI) listeners (Pichora-Fuller and Schneider, 1991; Moore, 1996; Gatehouse and Noble, 2004; Shinn-Cunningham and Best, 2008).

To compensate for the hearing impairment, aided hearing offers various advanced signal processing methods for digital noise suppression. Such noise reduction (NR) processing aims to reduce the level of interfering background noise to improve the signal-to-noise ratio (SNR; Chung, 2004; Dillon, 2012). Previous studies have shown that fast-acting NR processing reduces the listening effort in HI listeners and improves speech intelligibility at low SNRs (Wendt et al., 2017; Ohlenforst et al., 2018). The question is whether these improvements of speech intelligibility and reduced effort can also support selective auditory attention, i.e., improvements in a listener’s ability to selectively attend to one talker in a sound mixture.

Human neuroimaging studies, including electroencephalography (EEG), and magnetoencephalography (MEG), have demonstrated a strong attentional modulation of cortical responses by the substantial selective enhancement of neural responses to an attended talker *vs.* competing talker(s) during active listening (Ding and Simon, 2012a; Mesgarani and Chang, 2012; Power et al., 2012; Ding and Simon, 2013; Horton et al., 2013; Lakatos et al., 2013; Zion Golumbic et al., 2013; Horton et al., 2014; Kong et al., 2014; Evans et al., 2016; Fiedler et al., 2019). This stimulus–response correlation has been referred to as “neural representation,” “speech tracking,” or “neural entrainment” and reflects how strongly the speech envelopes of the individual talkers are represented in the M/EEG. The correlation reflects that concurrent competing talkers are encoded individually in brain areas associated with the auditory cortex. Moreover, the neural representation of the attended talker is higher compared to that of the ignored (competing) talker. That being said, the characterization of stimulus–response correlation using computational strategies plays a major role in investigating selective auditory attention.

Three inter-related computational strategies to characterizestimulus–response correlation have been proposed in the literature (Alickovic et al., 2019). First, stimulus reconstruction (SR; decoding) is aninverse mapping technique that finds a linear kernel, or response function, that best reconstructs (approximates) the speech envelope from the population of evoked neural responses. The correlation between reconstructed and actual speech envelopes—reconstruction accuracy—is often used as a metric to evaluate the fidelity of the neural representation of speech (Ding and Simon, 2012a; Mesgarani and Chang, 2012; O’Sullivan et al., 2015; Das et al., 2018; Aroudi et al., 2019). Second, a closely related strategy, termed neural response prediction (encoding), is a forward mapping technique that finds a linear kernel known as temporal response function (TRF) that best predicts the population of evoked neural responses from speech features (Lalor et al., 2009; Ding and Simon, 2012a, 2013; Di Liberto et al., 2015; Alickovic et al., 2016; O’Sullivan et al., 2019). Third, as an extension to these two strategies, a hybrid strategy combining the strengths (and the weaknesses) of encoding and decoding methods has also been proposed recently (de Cheveigné et al., 2018; Dmochowski et al., 2018; Iotzov and Parra, 2019).

Stimulus reconstruction has several advantages over the other methods to shed light on the neural mechanisms being targeted by hearing aids (HAs). First, it has been investigated in an extensive body of auditory literature in recent years. Second, the estimated neural representation of the speech envelope (reconstructed speech envelope) may uncover information about the perception and the processing of speech signals such as speech intelligibility (Ding and Simon, 2013; Di Liberto et al., 2018; Vanthornhout et al., 2018, Lesenfants et al., 2019; Verschueren et al., 2019), focused attention (O’Sullivan et al., 2015; Fuglsang et al., 2017), listener’s age (Presacco et al., 2016a, 2019; Brodbeck et al., 2018; Decruy et al., 2019), hearing loss (Presacco et al., 2019), and background noise level (Puvvada et al., 2017; Das et al., 2018; Khalighinejad et al., 2019).

Yet, to the best of our knowledge, there have not been attempts to evaluate the potential benefits of HAs equipped with NR schemes on selective auditory attention in complex settings that resemble everyday noisy social situations. The NR schemes in HAs may greatly benefit the HI listener’s ability to focus attention selectively by suppressing undesired background noise that is not the focus of attention. By improving SNR, fast-acting NR algorithms make the attended and the ignored talkers in front more audible, which could help the HA users to voluntarily switch attention between competing talkers while simultaneously suppressing background noise. This suggests that changes in NR processing in HAs may result in changes in the neural representation of speech envelopes.

Importantly, fast-acting active NR processing may enhance selective auditory attention by enhancing the strength of the neural representation of the foreground (attended and ignored) talkers while ensuring that the neural representation of the background noise is suppressed when compared to the inactive NR processing. Additionally, associated with the neural representation of the ignored talker and the ignored background noise, the net sum of the ignored acoustic scene outside of the primary focus of the listener’s attention (ignored talker and background noise in this study) should not be enhanced, i.e., either reduced or unchanged. Taken together, compared to inactive NR processing, fast-acting NR processing may be advantageous in that it may contribute to the improved “neural SNR,” which means that the fast-acting active NR processing may enhance the EEG responses to the attended talker while either reducing or keeping unchanged the EEG responses to the background noise and the ignored acoustic scene.

Therefore, to test the feasibility of quantifying the user benefits of NR schemes in HAs, inspired by a previous study with normal-hearing (NH) listeners (Das et al., 2018), we conducted a study in 22 HA users listening to continuous speech in a competing talker scenario. The NR schemes were tested in an “OFF” condition (inactive, where the NR feature was switched off in the HA) and an “ON” condition (active, where this feature was switched on in the HA) and under two different background noise levels to explore the influences of background noise level and noise reduction on the neural representation of speech. Sentences spoken by two talkers were presented from two different loudspeakers in front of the HI listener (i.e., in the foreground). Four loudspeakers, each playing a four-talker babble, were in the background. The HI listener was instructed to attend to one of the two talkers in the foreground while ignoring the other one and the background noise.

The NR scheme used in the present study improves the SNR of speech coming from the two foreground loudspeakers (i.e., both the attended and the ignored talkers) by predominately attenuating the background noise coming from the four background loudspeakers. The back-facing cardioid was used to determine the levels and the spatial locations of noise and speech sources in the environment. Then, a fast-acting combination of minimum variance distortion-less response beam-former (Kjems and Jensen, 2012), which is designed to improve SNR by using spatial filtering to attenuate noise between speech sources, and a single-channel Wiener post-filter (Jensen and Pedersen, 2015) was applied before amplification. If speech-like modulation is detected in one of the 16 frequency bands in a sound source in the environment, the system is expected to deactivate the beam-forming and post-filter NR in that band. This is done to prevent the attenuation of speech in the environment.

The NR scheme gives an overall SNR improvement of approximately +5 dB. For example, Ohlenforst et al. (2018) used a similar NR scheme and settings. In a test setup where a single target speaker was presented in two different types of background noise, the NR scheme provided SNR improvements up to +5.2 dB. The SNR improvement was further verified in technical measurements done in the specific test setup of the present study, which had two target talkers instead of one. The details of the technical measurements, together with the description of the NR scheme, are discussed in the “MATERIALS AND METHODS” section. Based on the approximately 5-dB SNR improvement provided by the NR scheme, the two SNR levels tested in this study also allowed us to investigate whether the SNR improvement provided by the NR scheme can be translated to a corresponding +5 dB of “neural SNR” improvement or, in other words, whether the NR-enhanced neural representation of the attended talker corresponds to a +5-dB shift in the reconstruction accuracy. This would be tested by comparing the test conditions +3 dB SNR with active NR (resulting in +8 dB SNR output from HA) and +8 dB SNR with inactive NR.

In combination with EEG-based speech reconstruction, this setup was designed to investigate whether NR processing in HAs affects the strength of the neural representation of different elements present in an acoustic scene and consequently supports selective auditory attention in HI listeners. We hypothesized that:

(H1) The active NR scheme would *enhance* the neural representation of the *attended speech* masked by a competing talker and noise compared to when the NR is inactive and across low and high SNRs.

(H2) The NR algorithm would *enhance* the neural representation of the *ignored speech* to a higher degree at a lower SNR than at a higher SNR.

(H3) The active NR processing would *reduce* the neural representation of the *background noise* across low and high SNRs.

(H4) The effect of the NR scheme on the neural representation of the attended speech would correspond to a +*5-dB shift* in the reconstruction accuracy *toward the higher SNR*.

## Materials and Methods

### Study Population

**FIGURE 1:**
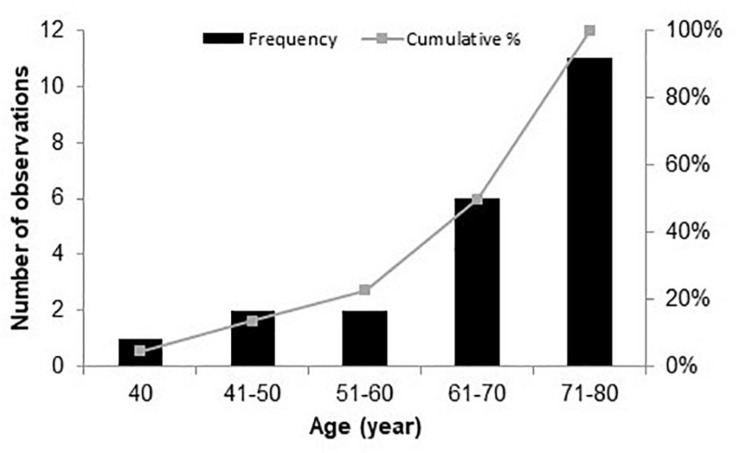
Frequency distribution of all participants by age.

**FIGURE 2:**
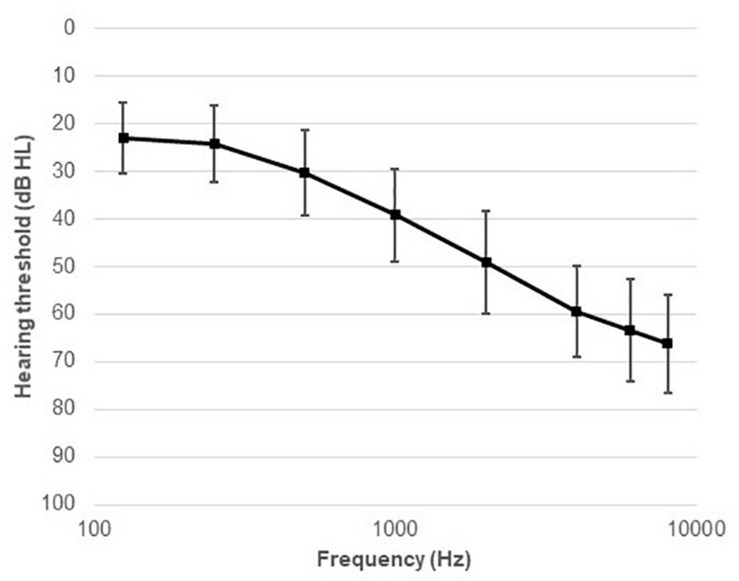
Average audiometric thresholds (mean and SD) across the entire frequency range measured (125–8,000 Hz).

Twenty-two native Danish speakers (11 males), aged between 40 and 80 years (mean age 67 years, SD = 11.2) years, were recruited from the Eriksholm Research Centre database (see Figure 1). All the participants were experienced HA users, with at least 4 months of HA usage. This criterion was chosen because it may take several weeks to months to get acclimatized to HAs (Munro, 2008; Dawes et al., 2014). The maximum difference between the left and the right ears’ audiometric thresholds (averaged between 500 and 4,000 Hz) was below 8 dB, and the thresholds at 500, 1,000, 2,000, and 4,000 Hz ranged from 33 to 58 dB hearing level (HL), with an average of 45 dB HL (see Figure 2). The inclusion criteria for the participants were mild to moderate sensorineural hearing threshold, normal or corrected-to-normal vision, and no history of neurological disorders, dyslexia, or diabetes mellitus.

The study was approved by the ethics committee for the capital region of Denmark (journal number H-1-2011-033). The study was conducted according to the Declaration of Helsinki, and all the participants signed a written consent prior to the experiment.

### Hearing Aid Fitting and Signal Processing

All the participants wore identical HA models, with two pairs of HAs fitted for each participant. The Voice Aligned Compression (VAC) amplification rationale (Le Goff, 2015) was applied in both pairs of HAs to compensate for hearing loss based on each individual’s hearing thresholds. The VAC amplification rationale is based on a curvilinear, wide, dynamic-range compression scheme with low-level compression knee points between 20 and 50 dB SPL (sound pressure level), depending on the frequency range and the hearing thresholds.

In one pair, the NR scheme was turned off (Le Goff, 2015). The HAs were set to the omnidirectional setting, with an added natural slight forward effect of the pinna. This mimics the pinna’s natural acoustic effect.

In the other pair, the NR scheme was activated. Technical measurements were done to verify the output SNR improvement in the HAs, which is defined as the difference between the input level of the environment (two target talkers and four background masker talkers) and HA output responses measured on a head and torso simulator (HATS). A pair of HAs was coupled to the HATS, and the output SNRs of the HAs were derived using the phase-inversion technique described in Björn and Olofsson (2004) with NR schemes ON and OFF. The articulation index weighted SNR improvements were 6.24 and 5.17 dB at +3 and +8 dB SNR for NR ON compared to that for NR OFF. These SNR improvements were within 1-dB difference from the maximum SNR improvement of what were reported by Ohlenforst et al. (2018).

### Speech Material

Danish news clips of neutral content were used for all speech streams. The two target streams were read by the same male and female talker for all 84 trials. The four-talker babble presented from each of the four loudspeakers in the back consisted of two female speakers and two male speakers, and none of the 16 streams were the same for each trial.

**FIGURE 3:**
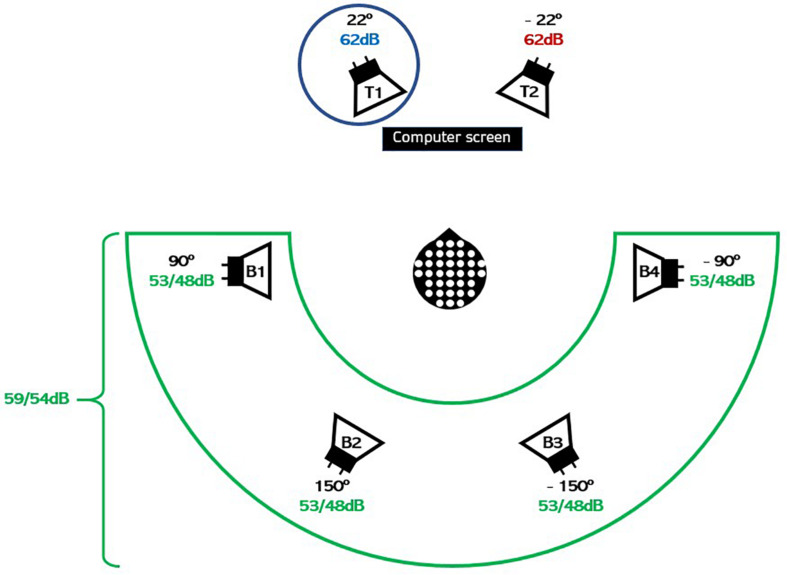
Schematic illustration of the experimental setup. The two target (competing) speakers in the foreground were positioned 44° (±22°) degrees apart and played news clips (continuous speech) at 62 dB. Each of the four masker (babble) speakers in the background played a four-talker babble summing up to either 59 or 54 dB. The circle around talker T1 shows that the HI listener in this trial was instructed to attend to talker T1 and hence to ignore the competing talker T2 and the four masker (babble) noises B1–B4. The HI listener was instructed to gaze at the computer screen during sound presentation, and neural responses were measured using EEG.

The background noise was delivered from four loudspeakers. Each loudspeaker played a different four-talker babble, leading to an overall effect of 16-talker surrounding babble to be ignored. Each babble stream was made from four unique single talkers—two females and two males—each reading a different news clip. The long-term average frequency spectrum of the babble noise was matched with the target talkers. The two target talkers were always presented at 65 dB, which was obtained by a fixed level of 62 dB SPL for each of the loudspeakers during the entire experiment, as indicated in Figure 3. Each of the maskers was presented at either 53 or 48 dB, leading to a total of 59 or 54 dB background SPL.

The SNR was defined as the ratio of signal power of the attended talker to the total signal power of the background noise, similar to that of Das et al. (2018). Hence, the SPL of the attended talker was raised either by +3 or +8 dB when compared to the background (4 × 4 talker babble) noise, which was tested at both NR schemes (OFF and ON) resulting in a 2 × 2 design. The particular SNR levels were chosen to create “real-world” listening conditions at different levels of difficulty. This level of background noise was defined as the long-term average sound level after eliminating pauses longer than 200 ms and applying root mean square equalization to the streams.

Stimuli were routed through a sound card (RME Hammerfall DSP multiface II, Audio AG, Haimhausen, Germany) and were played *via* loudspeakers (Genelec 8040A; Genelec Oy, Iisalmi, Finland).

### Study Design

Our experimental design was inspired by a previous study with NH listeners (Das et al., 2018). The participants were seated in a listening booth with controlled light conditions and with two target loudspeakers positioned at ±22° azimuth and four masker loudspeakers, each presenting a four-talker babble positioned at ±90° and ±150° azimuth, as illustrated in Figure 3. The task for the participant was to attend to one of the two talkers in the foreground while ignoring the other talker and the background babble noise. The participants were presented speech-on-speech from news clips obtained from Danish broadcasts (see section “Speech Material”).

**FIGURE 4:**
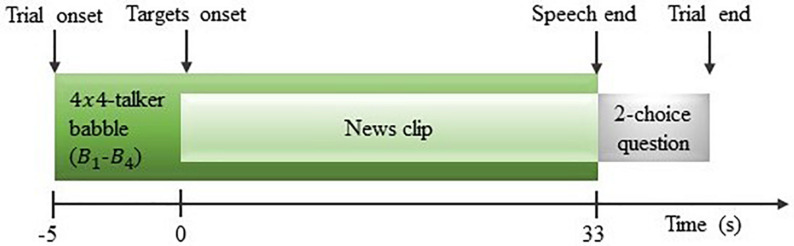
Illustration of a trial design.

A total of 84 trials were conducted, with four trials used for training and 80 trials used for testing and analysis. Each trial consisted of a short period of silence, 5 s of background noise followed by 33 s of simultaneous stimuli from all speakers, as illustrated in Figure 4. After each trial, a two-choice question about the content of the attended speech was presented to the participant to ensure sustained attention.

**FIGURE 5:**
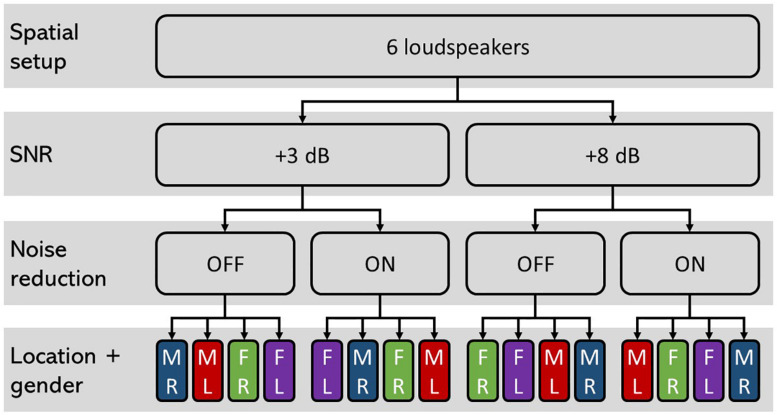
Schematic illustration of the study design. The four conditions “+3 dB OFF,” “+3 dB ON,” “+8 dB OFF,” and “+8 dB ON” were tested with 20 trials each. For each condition, the trials were randomized with five consecutive trials with attended speaker being “male right (MR),” “male left (ML),” “female right (FR),” and “female left (FL).” The order of these subconditions was randomized between conditions and participants and were applied to avoid bias toward one of the speakers or spatial awareness.

The trials were randomized into a block design, with 20 trials for each of the four experimental conditions: “+3 dB OFF,” “+3 dB ON,” “+8 dB OFF,” and “+8 dB ON.” The target talkers consisted of one male and one female talker. The male and the female talkers were presented by separate loudspeakers, and the gender and the position (left/right) were randomized. The 20 trials in each experimental condition (block) were divided into sub-blocks of five randomized consecutive trials for each of “male right,” “male left,” “female right,” and “female left,” as illustrated in Figure 5. This design was only used to obtain an equal number of attended speech streams and spatial distribution and not used for any further sub-analysis. Before each sub-block of five consecutive trials with the same attended talker, the participants were instructed on the screen to pay attention to the target on either the right or the left side and ignore the talker on the other side and the babble noise from behind. Additionally, 5 s of the attended speech from the to-be-attended spatial position was played to prepare the participant for the task. The participants were given a rest period after each of the four blocks. Two pairs of HAs were used, each pre-set with the two different noise reduction conditions. After each block, the HAs were removed and replaced with either the same pair of HAs (if the same NR condition was to follow), or the other pair of HAs (in cases where the other NR condition was to follow).

Each participant’s visit started with a training block in which the NR scheme was active (ON) and with one trial for each of the stimuli “male right,” “male left,” “female right,” and “female left” to ensure that the participants were confident with the experimental procedure.

### Neural Data Acquisition

Electroencephalography (EEG) data were recorded using a BioSemi ActiveTwo amplifier system (Biosemi, Amsterdam, Netherlands), with a standard cap including 64 surface electrodes mounted on the scalp according to the international 10–20 system. The cap included driven right leg and common mode sense electrodes, corresponding to the electrical reference, or “ground,” as reference for all other recording electrodes. To ensure stable connections to the scalp, the electrodes were prepared (and if necessary, supplied with additional gel) such that the offset voltage was stable and below 50 mV. The EEG signals were recorded with a sampling rate of 1,024 Hz.

### EEG-Based Envelope Reconstruction

Data analysis was done offline in Matlab R2018a (MathWorks) using the Fieldtrip toolbox (Oostenveld et al., 2011), mTRF Toolbox (Crosse et al., 2016), and custom-written scripts.

#### Neural Data Preprocessing

The signals picked up by each EEG electrode were epoched from 8 s before to 33 s after the onset of the two target (attended and ignored) speech stimuli. Then, 0.5-Hz high-pass filter, 95-Hzlow-pass filter, and 50-Hz notch filter were applied. Afterward, the data were downsampled to 512 Hz and referenced to the average of the two mastoid electrodes. The EEG channels with excessive noise were identified visually and removed. On average, 3.1 ± 0.8 channels were rejected. The noisy channels were interpolated from the surrounding clean EEG channels by using the nearest neighbor method in Fieldtrip (Oostenveld et al., 2011). The logistic Infomax independent component analysis algorithm (Bell and Sejnowski, 1995; Delorme and Makeig, 2004) was applied to calculate temporally independent components. The components were inspected visually and rejected if identified to clearly reflect artifacts caused by eye movements, eye blinks, muscle activity, heart beats, and single-channel noise. On average, 7.9 ± 3.6% of the components were rejected. One participant with excessively noisy data was removed from further analysis. Furthermore, due to technical problems, no data for one block of one participant was recorded. In a final step, the EEG signals were band-pass-filtered between 2 and 8 Hz (neural δ and θ bands) using a third-order Butterworth filter to extract the evoked neural activity representing slow temporal modulations in acoustic stimuli (Ding and Simon, 2012b; Zion Golumbic et al., 2013; O’Sullivan et al., 2015; Alickovic et al., 2019). Subsequently, data were downsampled to 128 Hz and segmented into trials of 33-s duration from 0 to 33 s relative to the onset of the attended speech stream.

#### Extraction of Acoustic Envelope

The acoustic envelope *U*_*i*_ was calculated by taking the absolute value of the analytic signal after a Hilbert transform of the raw sound signal at its original sampling rate (44.1 kHz), Butterworth low-pass filtering of the resulting waveform with a cutoff at 8 Hz (O’Sullivan et al., 2015), downsampling the result to match the sampling rate of the EEG data (128 Hz), and then separating the calculated envelopes into 33-s snippets to match their corresponding EEG trials. The envelope of the attended talker is referred to as the attended speech envelope (*U*_*A*_) and the envelope of the ignored talker is referred to as the ignored speech envelope (*U*_*I*_). In contrast, the envelope of the ignored background noise, comprising four different four-talker babbles *B*_1_, *B*_2_, *B*_3_, and *B*_4_ (see Figure 3), is referred to as the ignored background noise envelope (*U*_*IBN*_ = *U*_*B*_1_ + *B*_2_ + *B*_3_ + *B*_4__), and the envelope of the entire ignored acoustic scene, comprising both the ignored talker, and the ignored background noise (see Figure 3), is referred to as the ignored acoustic scene envelope (*U*_*IAS*_ = *U*_*I* + *B*_1_ + *B*_2_ + *B*_3_ + *B*_4__). These envelopes are used to design the neural models in section “Decoder Designs.”

#### Stimulus Reconstruction

To study the effects of NR processing on selective attention tasks in HI listeners, we used the method of stimulus (speech) reconstruction (SR). The SR estimates the unknown model response (in the literature, referred to as a linear kernel) function or decoder, which best relates evoked neural responses to (spectro-) temporal information in speech such as acoustic envelope. This allows a direct comparison between the reconstructed acoustic envelope and the envelope of the actual sound stream *via* Pearson’s *r* correlation coefficient, which reflects reconstruction accuracy—a measure of the fidelity of the neural representation of that acoustic envelope (Ding and Simon, 2012a; Presacco et al., 2016b).

With SR, the assumption is that the acoustic envelope can be explained as the convolution of the EEG signals with an unknown model response plus noise. Given the known acoustic envelope features and the measured EEG data, the model response is estimated in this study using dense linear regression, with an integration window set from 0 to 250 ms (O’Sullivan et al., 2015; Das et al., 2018). This design was chosen to capture the relevant temporal features in the EEG data which best correlate to each time point of the acoustic envelope to be reconstructed (see section “Supplementary Appendix A” for more details).

#### Decoder Designs

In order to quantitatively assess whether NR processing affects the neural representations of the sounds coming from different sound sources in an auditory scene, we estimated four different potential neural representations of sound elements in the multi-talker acoustic scene. To estimate the neural representations of the attended speech envelope, ignored speech envelope, ignored background noise envelope, and ignored acoustic scene envelope, four separate decoders were trained in a participant-specific manner for each experimental condition:

(1) The attended talker decoder trained on the responses to the attended talker.
(2) The ignored talker decoder trained on the responses to the ignored talker.
(3) The ignored noise decoder trained on the responses to the ignored background noise.
(4) The ignored acoustic scene decoder trained on the responses to the ignored acoustic scene consisting of the competing talker and background noise.

Each of the four decoding approaches was used to reconstruct the corresponding acoustic envelope whose similarity with the actual acoustic envelope was assessed *via* Pearson correlation coefficient *r*.

The attended talker decoder analysis tests the hypothesis that the active NR scheme enhances the neural representation of the attended speech. The ignored talker decoder analysis tests the hypothesis that the active NR processing enhances the neural representation of the ignored speech. The ignored noise decoder analysis tests the hypothesis that the active NR processing reduces the neural representation of the background noise. Finally, the ignored acoustic scene decoder analysis includes an additional exploration of the EEG responses to investigate whether the active NR processing either reduces or keeps unchanged (i.e., does not enhance) the neural representation of the ignored acoustic scene consisting of the competing talker and the background noise (i.e., sounds outside of the primary focus of the listener’s attention).

The four decoders were optimized separately for each experimental condition using leave-one-out (LOO) cross-validation (CV) while maximizing the Pearson’s *r* correlation between the reconstructed and the actual acoustic envelope (Crosse et al., 2016; Das et al., 2018). At the CV step *j*, the data from all trials but trial *j* were used to fit a model given in Eq. (2) in a per-trial manner, i.e., treating each trial as a single mapping problem, estimating one decoder for each training trial separately, and averaging across all but the *j*-th decoder. Then, the averaged decoder was used to reconstruct the envelope of the left-out *j*-th trial as in Eq. (4). The CV procedure was then repeated for all trials, thus allowing one to reconstruct one envelope for each trial [see O’Sullivan et al. (2015) and Alickovic et al. (2019) for more details on the “per-trial” training implementation]. These results were then grouped based on the experimental condition.

The regularization (ridge) parameter (see section “Supplementary Appendix A”) was set to a fixed value to produce the highest group mean LOO correlation (Pearson’s correlation *r*), i.e., to maximally correlate the reconstructed envelope with the acoustic envelope to be decoded across all participants and experimental conditions (λ = 10^5^).

### Statistical Analysis

All statistical analyses were conducted in R (version 3.6.1) using the R packages lme4 (Bates et al., 2014) and lmerTest (Kuznetsova et al., 2017) for fitting linear mixed models (LMMs). The function lmer was applied in this work to fit the LMMs to the data. We applied two-way LMM ANOVA to investigate the effect of the experimental conditions [NR, two types: inactive (OFF) *vs*. active (ON), SNR, and two levels: low (+3 dB) *vs*. high (+8 dB)] and their joint effect on the neural speech representations. We fitted one LMM ANOVA model for each of the four decoders, modeling reconstruction accuracy as a function of NR type and SNR, their corresponding interaction treated as fixed factors, and the participants treated as a repeated measure, i.e., random effects. The probability level was *p* < 0.05. *P*-values for the individual follow-up pairwise comparison of the NR processing settings [inactive (OFF) *vs*. active (ON)] at each SNR level were based on *t*-values and used Satterthwaite approximation for degrees of freedom.

## Results

We examined the effect of NR processing on the neural representation of the attended talker, ignored talker, ignored background noise, and ignored acoustic scene across different SNRs.

### Neural Representation of Attended Speech

**FIGURE 6:**
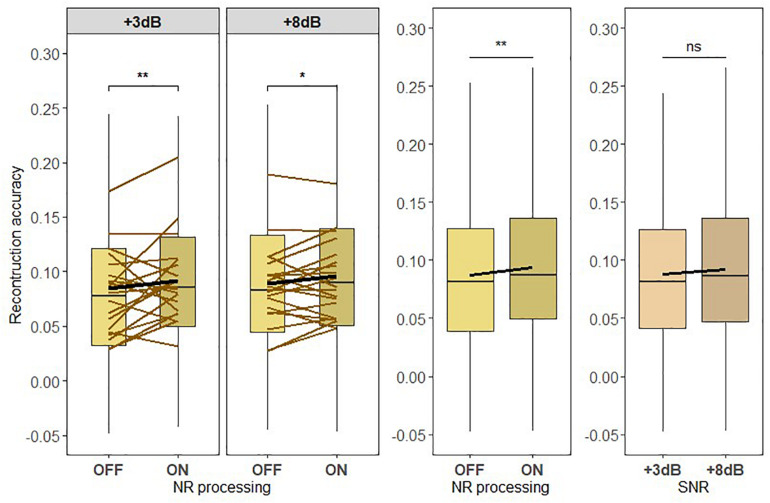
(H1) The active noise reduction (NR) scheme enhances the neural representation of attended speech to when the NR is inactive and across low and high signal-to-noise ratio (SNR) levels. Boxplots showing the reconstruction accuracy values of the attended speech envelope reconstructed from the attended talker decoder for two SNR levels tested with NR scheme inactive (OFF) or active (ON; left column) for global averages across NR schemes (middle column) and for global averages across SNR levels (right column). A significant main effect of NR scheme setting, indicating the enhanced neural representation of the attended speech when NR scheme was active, was observed. Significant differences in reconstruction performances between inactive and active NR in the pairwise comparison at each SNR level were observed. The horizontal lines in yellow denote single participants and the horizontal lines in black depict predictions of linear mixed models. The black horizontal line in the boxplot denotes the mean reconstruction accuracy of the attended speech envelope and the box indicates the upper and the lower quartiles, with the vertical lines representing the minimum and the maximum reconstruction accuracy values. The asterisks indicate significant differences (*p < 0.05, **p < 0.01); ns, not significant.

To test the hypothesis H1, we used the attended talker decoder to reconstruct the attended speech envelope to assess the strength of the neural representation of attended speech across four different listening conditions (see Figure 6). As shown in Figure 6, the average reconstruction accuracy improves when the NR processing is active. The LMM ANOVA of the reconstruction accuracy *r*_*A*_ with main factors of SNR and NR found a significant effect for NR [*F*_419_ = 9.2783, *p* = 0.0024]. No significant effect was found for SNR [*F*_419_ = 0.2849, *p* = 0.59369]. Furthermore, no significant effect for interaction between SNR and NR [*F*_419_ = 0.2558, *p* = 0.6131] was found, suggesting that NR was equally effective at enhancing the reconstruction accuracy *r* at both SNR levels. A follow-up pairwise comparison for each SNR level confirmed that the significant effect of NR processing was present at both SNR levels [+ 3dB:*p* = 0.0092, + 8dB:*p* = 0.0366]. In addition to this, we also tested whether the active NR scheme shifts the reconstruction accuracy by approximately 5 dB toward the higher SNR. Figure 6 shows that the average reconstruction accuracy is higher at “+3 dB ON” condition than at “+8 dB OFF” condition. However, the *post hoc* pairwise comparison did not reveal the significant difference in the reconstruction accuracy values between these two conditions [*p* = 0.0782]. Given the non-significant interaction between SNR and NR, these results could be seen to suggest that the NR-enhanced neural representation also corresponds to a +5-dB shift (even though the SNR improvement from HA is relatively less, which is 3.6 dB). These results validated that a NR scheme can significantly improve the neural representation of attended speech in, for the HI individuals, challenging sound environments and that this improvement could correspond to the shift of 5 dB toward the higher SNR.

### Neural Representation of Ignored Speech

**FIGURE 7:**
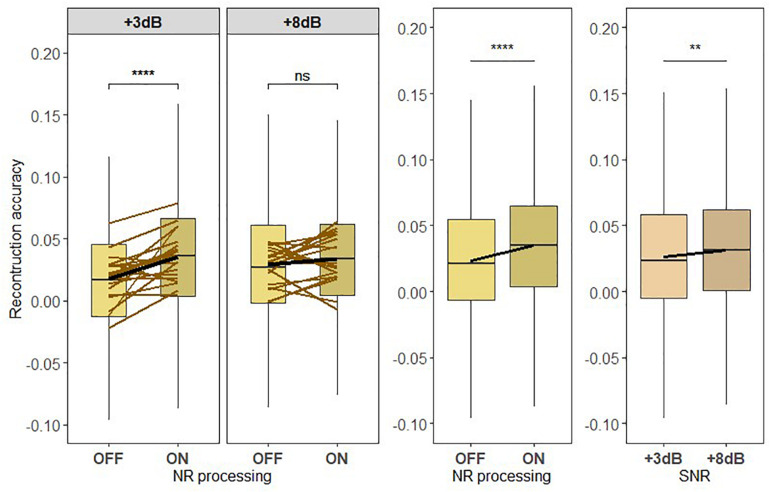
(H2) The NR algorithm enhances the neural representation of the ignored speech to a higher degree at a lower signal-to-noise ratio (SNR) level than at a higher SNR. Boxplot showing the reconstruction accuracy value of the ignored speech envelope reconstructed from the ignored talker decoder for two SNR levels tested with noise reduction (NR) scheme inactive (OFF) and active (ON; left column) for global averages across NR schemes (middle column) and for global averages across SNR levels (right column). A significant main effect of NR, indicating the enhanced neural representation of the ignored speech when NR scheme was turned on, and a significant main effect of SNR were observed. Significant differences in reconstruction performances were seen between inactive and active NR in the pairwise comparison at lower SNR (+3 dB). The horizontal lines in yellow denote single participants and the horizontal lines in black depict predictions of linear mixed models. The black horizontal line in the boxplot denotes the mean reconstruction accuracy of the ignored speech envelope and the box indicates the upper and the lower quartiles, with the vertical lines representing the minimum and the maximum reconstruction accuracy values. The asterisk indicates significant differences (**p < 0.01, ****p < 0.0005); ns, not significant.

Prior to testing H2, we investigated how the neural representation of the ignored talker compared to the neural representation of the attended talker (see Figure 7). We applied a one-way LMM ANOVA to test the effect of attention on the neural representation of speech. In this model, reconstruction accuracy was treated as a dependent measure, attention was treated as a fixed effect, and the participants were treated as a random measure, i.e., random effects. The LMM ANOVA revealed a significant main effect of attention (*F* = 942.5845, *p* < 2.2e-16), which confirms that the attended talker is more strongly represented in the brain than the ignored talker.

To test the hypothesis H2, we investigated whether the neural representation of the ignored speech envelope, reconstructed using the ignored talker decoder, depended on NR scheme and SNR level. Figure 7 shows the reconstruction performances of the ignored speech envelope for inactive and active NR schemes for the two SNR levels. The reconstruction accuracy for the ignored speech envelope was modulated by SNR [*F*_419_ = 6.767, *p* = 0.0094] and NR [*F*_419_ = 27.816, *p* < 0.0005], with an interaction between SNR and NR [*F*_419_ = 10.966, *p* = 0.002], indicating that the reconstruction accuracy (neural representation) of the ignored speech is affected by the differences in listening difficulty (SNR) and the NR processing algorithm. The interaction effect between NR scheme and SNR level implies that the NR effect of the reconstruction accuracy for the ignored speech envelope is more prominent in the difficult listening situation at +3 dB, as illustrated in Figure 7. A follow-up pairwise comparison revealed significant differences in reconstruction accuracy (neural representation) of the ignored speech between NR scheme settings at lower SNR (*p* < 0.0005), but not at higher SNR (*p* = 0.1621).

### Neural Representation of the Ignored Background Noise

**FIGURE 8:**
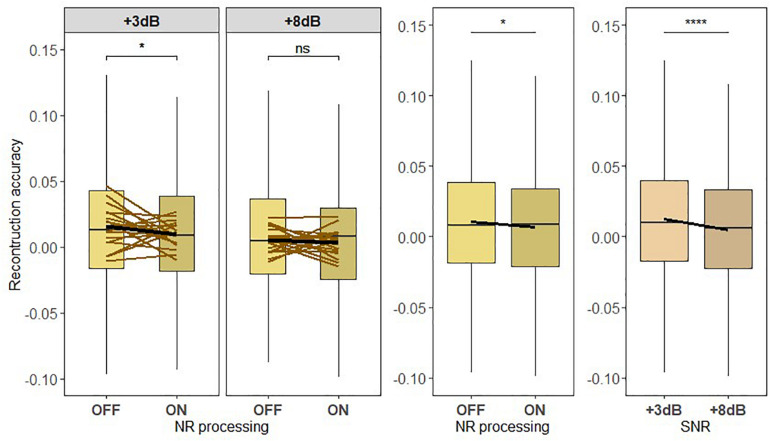
(H3) The active NR processing reduces the neural representation of the background noise across low and high signal-to-noise ratio (SNR) level. Boxplot showing the reconstruction accuracy values of the ignored background noise envelope reconstructed from the ignored noise decoder for two SNR levels tested with noise reduction (NR) scheme inactive (OFF) or active (ON; left column) for global averages across NR schemes (middle column) and for global averages across SNR levels (right column). A significant main effect of NR, indicating the reduced neural representation of the ignored background noise when NR scheme was turned on, and a significant main effect of SNR were observed. A significant difference in reconstruction performances between inactive and active NR in the pairwise comparison at lower SNR (+3 dB) was observed. The horizontal lines in yellow denote single participants and the horizontal lines in black depict predictions of linear mixed models. The black horizontal line in the boxplot denotes the mean reconstruction accuracy of the ignored background noise envelope and the box indicates the upper and the lower quartiles, with the vertical lines representing the minimum and the maximum reconstruction accuracy values. The asterisk indicates significant differences (*p < 0.05, ****p < 0.0005); ns, not significant.

To test hypothesis H3, Pearson correlation between the neural representation of the ignored background envelope estimated from the ignored noise decoder and the actual ignored speech envelope was calculated for each participant and experimental condition (see Figure 8). The results obtained from the analysis are shown in Figure 8. There was a significant main effect of NR scheme setting [*F*_419_ = 6.214, *p* = 0.013] and correlations were overall lower when the NR scheme was active. The LMM ANOVA also revealed a significant main effect of SNR [*F*_419_ = 21.3262, *p* < 0.0005] and correlations were overall lower when the SNR was increased. There was no significant interaction effect between NR scheme and SNR [*F*_419_ = 1.1101, *p* = 0.2922]. Follow-up pairwise comparisons of the two NR scheme settings (OFF or ON) were applied at the two SNR levels. Significant differences in correlations were observed in the follow-up pairwise comparison between the NR schemes at lower SNR (+3 dB; *p* = 0.0351), but not at higher SNR (+8 dB; *p* = 0.5359). These results revealed a significant reduction of the neural representation of background noise when the NR scheme was turned on.

### Neural Representation of the Ignored Acoustic Scene

**FIGURE 9:**
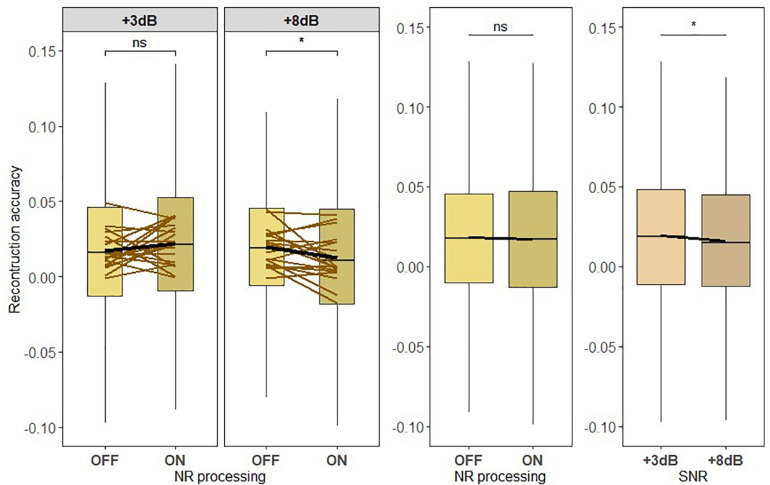
The active NR processing does not enhance the neural representation of the ignored acoustic scene consisting of the ignored talker and background noise. Boxplot showing the reconstruction accuracy values of the ignored acoustic scene envelope reconstructed from the ignored acoustic scene decoder for two signal-to-noise ratio (SNR) levels tested with noise reduction (NR) scheme inactive (OFF) or active (ON; left column) for global averages across NR schemes (middle column) and for global averages across SNR levels (right column). A significant main effect for SNR and an interaction between SNR and NR were observed. The results showed no significant main effect of NR. A significant difference in reconstruction performances between inactive and active NR in the pairwise comparison at higher SNR (+8 dB) was observed. The horizontal lines in yellow correspond to each participant. The horizontal lines in yellow denote single participants and the horizontal lines in black depict predictions of linear mixed models. The black horizontal line in the boxplot denotes the mean reconstruction accuracy of the ignored acoustic scene envelope and the box indicates the upper and the lower quartiles, with the vertical lines representing the minimum and the maximum reconstruction accuracy values. The asterisk indicates significant differences (*p < 0.05); ns, not significant.

Finally, we exploratorily investigated the neural representation of the ignored acoustic scene consisting of the ignored talker and the background noise. The neural representation of the ignored acoustic scene envelope was estimated using the ignored acoustic scene decoder (see Figure 9). Figure 9 shows the correlation values reflecting the reconstruction performances for each NR scheme setting tested at each of the SNR conditions. We found a significant main effect of SNR [*F*_419_ = 4.3828, *p* = 0.03654]. However, no significant effect for NR scheme was observed [*F*_419_ = 0.4205, p = 0.5168], showing that the NR scheme did not significantly enhance the neural representation of the ignored acoustic scene. A significant interaction was also observed between SNR and NR, indicating that the NR dependency of the reconstruction performance differs according to the SNR level [*F*_419_ = 7.7392, *p* = 0.0055]. A pairwise *post hoc* comparison for each noise condition revealed a significant difference between NR scheme settings at +8 dB SNR (*p* = 0.0153915), indicating a significantly reduced neural representation of the ignored acoustic scene. Hence, these results suggest that an active NR scheme should not enhance the ignored acoustic scene.

## Discussion

In this study, we systematically studied the effect on NR and SNR on neural speech representation during selective attention by means of SR methods applied to EEG responses. Taken together, we showed that active NR enhanced the neural representation of both the attended and the ignored speaker and reduced the neural representation of background noise, while the net sum of the ignored acoustic scene (ignored talker and background babble) was not enhanced. In addition, the condition with low SNR at +3 dB had increased neural background noise suppression as compared to the condition with high SNR at +8 dB. Hence, the overall neural representation of the foreground was enhanced as compared to the background noise during an active NR scheme. These findings were consistent across two SNR levels. Thus, these results suggest that the NR algorithms in HAs play an important role in enhancing the neural representation of speech and reducing the neural representation of background noise in HI listeners during a selective auditory attention task.

### Contribution of NR Processing and SNR on Neural Representation of Speech

#### Attended Speech

The neural representation of the attended talker was significantly affected by the levels of NR scheme (ON *vs.* OFF) and SNR conditions. Our main finding was that the neural representation of attended speech was enhanced when the NR scheme was active as compared to inactive. Furthermore, as a complementary finding, our results revealed that the changes in SNR level were accompanied by changes in the reconstruction accuracy of attended speech in HI listeners. As expected, a lower SNR produced lower reconstruction accuracy, while a high SNR produced higher reconstruction accuracy of attended speech. This observation is in line with a recent study with NH listeners (Das et al., 2018). Next to Das et al. (2018), our results are in line with the results reported by Decruy et al. (2020), showing that reconstruction fidelity increases with increasing speech understanding in both NH and HI participants. Furthermore, this association between increased reconstruction fidelity with increased SNR and *vice versa* is also consistent with the results reported in recent studies with older clinically NH listeners (Presacco et al., 2016a, b) and older HI listeners (Presacco et al., 2019). Here, for the first time, we showed that the decline in reconstruction accuracy with decreasing SNR can be corrected with an active NR scheme in HAs. Interestingly, we also found that the neural representation of the attended speech envelope at lower (+3 dB) SNR was shifted by approximately 5 dB toward the higher (+8 dB) SNR when the NR scheme was turned on. In this context, it may be worth noting that previous studies targeting attention gain used the weighting to a given stream that was roughly 10 dB higher when it was attended compared to when another sound was attended (Choi et al., 2013). As hypothesized (H1 and H4), our results hence suggest that the active NR scheme helps the HI listeners to selectively attend to the talker of most importance to them in challenging listening environments.

#### Ignored Speech

The neural representation of the ignored talker was enhanced during active NR processing, and this enhancement was more prominent at a lower SNR. As the NR algorithm should suppress background sounds while still providing audibility of sound sources in front of the listener, this demonstrates the benefit of active NR to enable the HA user to switch attention between competing talkers. This finding is consistent with a recent study which reported that the neural representation of ignored speech was significantly affected by the acoustic environment (Fuglsang et al., 2017). Fuglsang and colleagues found that the reconstruction accuracy of ignored speech was higher in an anechoic condition compared to mildly and highly reverberant conditions. In addition, this finding is also consistent with an earlier study that tested the perceptual load theory (Lavie, 1995) of selective auditory attention and found that reduced perceptual load leads to increased neural processing of ignored speech streams as reflected by an increased cortical segregation of the ignored speech streams (Hausfeld et al., in review). As hypothesized (H2), these findings indicate that the acoustic environment may influence the neural representation by reducing the reconstruction fidelity of the ignored talker in more complex acoustic scenes. Our study took this a step further by examining the effect on neural representations of the ignored talker in the case of varying background noise levels obtained by NR schemes in HAs.

#### Background Noise

As hypothesized (H3), the active NR processing reduced the overall neural representation of the background noise as compared to inactive NR. This result was observed by reconstructing the envelope of unsegregated background noise sounds rather than reconstructing the envelope for each background noise object and then averaging them. This approach was based on findings in recent studies suggesting that the background streams are more accurately represented as a single unsegregated background noise object rather than separated noise objects (Puvvada et al., 2017; Hausfeld et al., 2018).

Hence, the activation of a NR scheme suppressed the background noise features in the evoked neural responses in HI listeners. This finding confirms that NR processing plays a role in reducing background noise in the cortex and thus may lead the HA user to find the background noise less troubling (Dillon, 2012). This reduction is likely due to the NR algorithms applied in this study, which attenuates background noise by combining beam-former (Kjems and Jensen, 2012) and a single-channel Wiener post-filter (Jensen and Pedersen, 2015).

#### Ignored Acoustic Scene

An additional exploration of the response shows that fast-acting NR algorithms enhance the neural representation of the attended speaker while ensuring that the neural representation of the ignored acoustic scene is not enhanced, i.e., either reduced or unchanged. We observed that the neural representation of the ignored acoustic scene was reduced at high SNR levels with active NR, while it was unchanged between NR settings at low SNR level. This result was obtained by reconstructing the envelope of the unsegregated ignored acoustic scene based on previous findings (Puvvada et al., 2017; Hausfeld et al., 2018).

### Neural Speech Representation as a Tool to Evaluate HA Settings

The experimental design introduced in this study allowed us to analyze the effect of NR processing on cortical responses to both the foreground (competing talkers) as well as the background noise across two different SNR levels using SR method. We hypothesized that, under challenging listening conditions, HAs can alleviate the impact of background noise while providing adequate access to foreground streams during selective auditory attention by applying high-end NR signal processing algorithms. It therefore appears reasonable to use the SR to objectively assess how well the talkers of interest in the foreground can be enhanced and the noise in the background suppressed in the brain with different HA settings. In our previous studies, we showed that NR scheme processing had a significant positive effect on sentence recognition and listening effort measured by peak pupil dilation (Wendt et al., 2017, 2018; Ohlenforst et al., 2018). We thus designed this experiment to see whether the NR scheme processing also has a significant positive effect on neural speech representation and consequently on selective auditory attention.

Our results indicate that the NR processing alters the neural representations of sounds present in complex acoustic scenes across different SNRs, demonstrating the role of NR algorithms in natural sound environments. Crucially, we showed that the NR algorithm applied in this study enhanced the neural representations of attended and ignored talkers and reduced the neural representation of ignored background noise. We also give the evidence that the active NR corresponds to a shift in the neural representations of sound elements from 5 dB toward a higher SNR. Our results indicate that some aspects of selective auditory attention in HI listeners can be improved independent of SNR. Our study confirmed that the currently tested NR schemes enhance the neural representation of both the attended and the ignored talkers, leading to higher detail and audibility of the speech streams. This may ease selective auditory attention to enable the listener to attend to the speech of interest and switch attention if desired.

The approach to quantitively assess the effect of NR processing on the representation of speech in the brain using SR applied to EEG responses may, in the future, provide a valuable tool to explore the user benefits of different HA settings. Whether age and degree of hearing loss would further influence the improved neural representations with a given HA setting should be explored. Determining how improved neural representations are linked to different perceptual benefits (such as improved speech perception and subjective listening abilities) would also have implications in prescribing amplification as well as providing insights into the design of new HA technology. With this approach in hand, we can now begin answering open questions concerning the benefits of HAs in solving the cocktail party problem in more realistic listening scenarios with continuous speech.

In particular, the goal of our future studies will be to reveal how specific hearing-loss-related deficits in neural processing at various hierarchical levels in the auditory system (Puvvada and Simon, 2017; O’Sullivan et al., 2019; Brodbeck et al., 2020) lead to failures of selective attention (i.e., ability to separate and follow the target sound over time) and whether HAs can compensate for these deficits. Such an understanding of the links between specific neural deficits and their perceptual consequences may help identify HI listeners who have particular difficulties in deploying selective attention in complex acoustic situations. Toward that goal, speech tracking (i.e., neural representation of speech) measures hold a potential to delineate how higher-order acoustic, phonetic, and linguistic speech features are represented and integrated at different levels of the auditory system and to understand the role played by individual auditory regions during selective attention in unaided and aided conditions.

### Limitations of the Study Design

A major limitation of the experimental design proposed in this study is the restriction on randomization. Overall, four experimental conditions (“+3 dB OFF,” “+3 dB ON,” “+8 dB OFF,” and “+8 dB ON”) were tested in a randomized block design rather than a completely randomized design, which would allow us to avoid practice effects. This was decided for two reasons. First, each HI participant had a pair of HAs fitted for inactive (OFF) and active (ON) NR scheme settings. The HAs were removed and replaced at the end of each experimental condition, whether or not the NR condition actually changed, which would not be possible in a fully randomized design. Second, altering the experimental condition after each trial (i.e., every 38 s) may induce surprise and fatigue to the participants. Furthermore, the completely randomized design could have emphasized the two SNR conditions as the background noise level changed between SNR conditions, and hence the 5-s background noise prior to the onset of target talkers could have primed the participant on task difficulty. The randomization between gender and spatial position of the attended talker was likewise applied in a randomized block design to allow the HI participant to focus on the listening task rather than on the task instructions. This issue may be partially addressed by instructing the HI participant to attend to the same talker throughout all 80 trials. Nevertheless, a randomized block design was preferred in this study for the reasons that were described earlier.

A related limitation specific to the present study is the spatial separation of the target (attended and ignored) talkers in the foreground. The two target talkers were positioned 44° apart, which was the maximum possible separation due to dimensions of the listening booth. Previous studies have shown that the separation of the speakers affects the mean decoding accuracy, with an advantage of having larger angular separation between the two target talkers due to spatial release from masking (Kidd et al., 1998; Gary and Litovsky, 2011; Das et al., 2018). However, since we were testing NR schemes in HAs with spatial filtering and Wiener filtering to attenuate sounds originating behind the listener, constraints apply in the spatial separation. Therefore, we believe that the separation of 44° is an appropriate design for the study. Additionally, the primary goal of the present study was to investigate the effects of NR and SNR, and hence the speaker separation effect was kept constant between the experimental conditions.

While we provide strong evidence for the benefits of HA signal processing on the neural representation of speech during selective auditory attention, another limitation of this study is that we did our analyses on a population basis, and thus we did not explore the role of age or other factors that may affect selective attention. In our current sample, there is a considerable spread in age (half of the participants were 70 years or older, while some participants were well below 60 years). Future studies are warranted to further investigate whether the benefits of the hearing aid signal processing vary across age and whether such benefits can be extended to hearing aid users with more severe hearing loss.

Finally, content-related question-and-answer procedure may not be suitable to ensure sustained attention to one of the multiple talkers. In particular, the HI participants were asked a two-choice question after each trial about the content of the attended speech (i.e., random snippet of the attended news clip). For each question, there were two alternatives options: “True” or “False.” These questions served only to secure the sustained attention to one of the two competing talkers in the foreground (news clips). As the questions were only on a short snippet of the attended news clip, the participants may have heard the content but could not recall the correct answer. Similarly, the participants might have guessed the correct answer without attending or hearing the content of the news clip. Due to these uncertainties, we did not correlate our findings to behavioral performance.

## Conclusion

We demonstrated that a noise reduction scheme in commercial HAs can help listeners to focus attention in challenging listening environments by attenuating the neural representation of unwanted background noise and enhancing the neural representation of foreground sounds. Overall, we compared the reconstruction accuracy (a metric to evaluate the fidelity of the neural speech representation) for two different SNRs and two different noise reduction schemes. We found an improvement in the neural representation of attended speech with an active NR scheme, shifting the reconstruction accuracy by approximately 5 dB toward the higher SNR, indicating a selective enhancement of neural responses to the attended talker. In addition, a main effect of NR processing on the reconstruction accuracy of background noise was observed, indicating the suppression of neural responses to background noise. Furthermore, we found that the neural representation of the ignored speech envelope was also modulated by noise reduction scheme and was mostly enhanced in the conditions with more background noise, indicating the enhancement of the neural responses to the ignored talker and the improved ability to switch attention at will. Taken together, these results confirm that NR processing in HAs serves to support selective auditory attention in natural listening.

## Data Availability Statement

There are ethical restrictions on sharing the data set. The consent given by participants at the outset of this study did not explicitly detail sharing of the data in any format; this limitation is keeping with EU General Data Protection Regulation, and is imposed by the Research Ethics Committees of the Capital Region of Denmark. Due to this regulation and the way data was collected with low number of participants, it is not possible to fully anonymize the dataset and hence cannot be shared. As a non-author contact point, data requests can be sent to Claus Nielsen, Eriksholm research operations manager at clni@eriksholm.com.

## Ethics Statement

The studies involving human participants were reviewed and approved by Journal number H-1-2011-033. The patients/participants provided their written informed consent to participate in this study.

## Author Contributions

TL, EA, CG, DW, and RH contributed to study concept, hypothesis generation, and design of the experiment. EA, CG, and RH recorded the data. EA analyzed the data. EA, TL, CG, EN, LF, and DW interpreted the data. EA and CG drafted the manuscript. EA, CG, TL, EN, LF, and DW read the manuscript and provided critical revision. All authors contributed to the article and approved the submitted version.

## Conflict of Interest

EA, TL, DW, LF, RH, and CG were employed by company Eriksholm Research Centre, Oticon A/S, Snekkersten, Denmark. EN was employed by company Oticon A/S, Smørum, Denmark.
