# Supplementary material for: Neural Representation Enhanced for Speech and Reduced for Background Noise With a Hearing Aid Noise Reduction Scheme During a Selective Attention Task
Source: Front Neurosci. 2020 Sep 10;14:846. doi: 10.3389/fnins.2020.00846 (PMC7533612; doi:10.3389/fnins.2020.00846)
Supplement: Supplementary file 1 [file Data_Sheet_1.docx]

1. **STIMULUS RECONSTRUCTION**

The SR algorithm used in this study was performed using dense ($l_{2}$-regularized) linear regression (LR) (O'Sullivan, et al., 2015; Crosse, et al., 2016; Wong, et al., 2018; Alickovic, et al., 2019). In the training phase, the SR procedure involves estimating a liner spatio-temporal decoder(s)$A_{i}$, consisting of different weights $a_{ijn}$ assigned to different EEG channels$j$ and different time lags$n$, that best maps EEG signals (evoked neural responses) to the acoustic envelope. Mathematically, this mapping can be described as

|  | $u_{i}\left[ k \right]= \sum_{j=1}^{n_{y}} A_{ij}\left( q \right)y_{j}\left[ k \right]+\varepsilon_{i}[k]$ | (1) |
| --- | --- | --- |

where acoustic envelope $u_{i}$ (see Section 2.6.2) is explained as a sum of filtered EEG signals$y_{j}$ plus additive noise $\varepsilon_{i}$ to account for measurement errors and model imperfections. Here, $i=\left\{ A, I,IBN, IAS \right\}$ denotes the acoustic envelope type to be reconstructed (see Section 2.6.2), $k$is a time instant, $q$ is a shift operator defined by $q^{n}y_{j}\left[ k \right]=y_{j}\left[ k+n \right]$, $A_{ij}\left( q \right)=a_{ij0}+a_{ij1}q^{1}+\cdots+a_{ijn_{a}}q^{n_{a}}$ and denotes a linear kernel mapping the $j$-th EEG channel to the $i$-th acoustic envelope, $n_{y}$ denotes the number of EEG channels and $n_{a}$ denotes the integration window - the number of time lags used to estimate the decoder and to reconstruct one single point of the acoustic envelope $u_{i}$. The decoding model in (1) can more conveniently be expressed in matrix-vector form as

|  | $U_{i}\mathcal{= H}\left( Y \right)A_{i}+E_{i}$ | (2) |
| --- | --- | --- |

where${[Y_{j}]}_{k}=y_{j}\left[ k \right]$,${[U_{i}]}_{k}=u_{i}\left[ k \right]$, ${[\mathcal{H}\left( Y \right)]}_{j}=\mathcal{H}\left( Y_{j} \right)$ and $\mathcal{H}\left( Y_{j} \right)$is a Henkel matrix defined as$\left[ \mathcal{H}\left( Y \right) \right]_{kn}= y_{j}\left[ n_{a}+k-n \right]$ containing sample-wise time-lagged replications of $j$-th EEG channel. In this study, the entire decoder $A_{i}$for all $n_{y}$EEG electrodes ($n_{y}=64$) was simultaneously obtained using dense LR to minimize the mean squared difference between reconstructed and actual speech envelopes as

|  | $A= {{\mathcal{(H}\left( Y \right)}^{T}\mathcal{H}\left( Y \right)+\lambda I)}^{-1}{\mathcal{H}\left( Y \right)}^{T}U_{i}$ | (3) |
| --- | --- | --- |

Dense LR provides a regularization parameter $\lambda$to prevent overfitting. See (Alickovic, et al., 2019) for further details.

Next, given such a decoder$A_{i}$, the objective is to reconstruct the acoustic envelope $\hat{U}_{i}^{n}$ of the sound source $i$ from the new EEG data$Y^{n}$, unseen by the model in the training phase and to compare the quality of these reconstructions across different experimental conditions. The acoustic envelope $\hat{U}_{i}^{n}$ can be reconstructed as

|  | $\hat{U}_{i}^{n}=\mathcal{H}\left( Y^{n} \right)A_{i}$ | (4) |
| --- | --- | --- |

The reconstruction accuracy can be described as the Pearson’s correlation $r$ between the reconstructed acoustic envelope$\hat{U}_{i}^{n}$and the corresponding actual acoustic envelope$U_{i}^{n}$, and represents the quantitative measure of the fidelity of the neural representation of the sound.
